# Music in Our Ears: The Biological Bases of Musical Timbre Perception

**DOI:** 10.1371/journal.pcbi.1002759

**Published:** 2012-11-01

**Authors:** Kailash Patil, Daniel Pressnitzer, Shihab Shamma, Mounya Elhilali

**Affiliations:** 1 Department of Electrical and Computer Engineering, Center for Language and Speech Processing, Johns Hopkins University, Baltimore, Maryland, United States of America; 2 Laboratoire Psychologie de la Perception, CNRS-Université Paris Descartes & DEC, Ecole normale supérieure, Paris, France; 3 Department of Electrical and Computer Engineering and Institute for Systems Research, University of Maryland, College Park, Maryland, United States of America; University of California at Berkeley, United States of America

## Abstract

Timbre is the attribute of sound that allows humans and other animals to distinguish among different sound sources. Studies based on psychophysical judgments of musical timbre, ecological analyses of sound's physical characteristics as well as machine learning approaches have all suggested that timbre is a multifaceted attribute that invokes both spectral and temporal sound features. Here, we explored the neural underpinnings of musical timbre. We used a neuro-computational framework based on spectro-temporal receptive fields, recorded from over a thousand neurons in the mammalian primary auditory cortex as well as from simulated cortical neurons, augmented with a nonlinear classifier. The model was able to perform robust instrument classification irrespective of pitch and playing style, with an accuracy of 98.7%. Using the same front end, the model was also able to reproduce perceptual distance judgments between timbres as perceived by human listeners. The study demonstrates that joint spectro-temporal features, such as those observed in the mammalian primary auditory cortex, are critical to provide the rich-enough representation necessary to account for perceptual judgments of timbre by human listeners, as well as recognition of musical instruments.

## Author Summary

Music is a complex acoustic experience that we often take for granted. Whether sitting at a symphony hall or enjoying a melody over earphones, we have no difficulty identifying the instruments playing, following various beats, or simply distinguishing a flute from an oboe. Our brains rely on a number of sound attributes to analyze the music in our ears. These attributes can be straightforward like loudness or quite complex like the identity of the instrument. A major contributor to our ability to recognize instruments is what is formally called ‘timbre’. Of all perceptual attributes of music, timbre remains the most mysterious and least amenable to a simple mathematical abstraction. In this work, we examine the neural underpinnings of musical timbre in an attempt to both define its perceptual space and explore the processes underlying timbre-based recognition. We propose a scheme based on responses observed at the level of mammalian primary auditory cortex and show that it can accurately predict sound source recognition and perceptual timbre judgments by human listeners. The analyses presented here strongly suggest that rich representations such as those observed in auditory cortex are critical in mediating timbre percepts.

## Introduction

A fundamental role of auditory perception is to infer the likely source of a sound; for instance to identify an animal in a dark forest, or to recognize a familiar voice on the phone. Timbre, often referred to as the color of sound, is believed to play a key role in this recognition process [1]. Though timbre is an intuitive concept, its formal definition is less so. The ANSI definition of timbre describes it as that attribute that allows us to distinguish between sounds having the same perceptual duration, loudness, and pitch, such as two different musical instruments playing exactly the same note [2]. In other words, it is neither duration, nor loudness, nor pitch; but is likely “everything else”.

As has been often been pointed out, this definition by the negative does not state what are the perceptual dimensions underlying timbre perception. Spectrum is obviously a strong candidate: physical objects produce sounds with a spectral profile that reflects their particular sets of vibration modes and resonances [3]. Measures of spectral shape have thus been proposed as basic dimensions of timbre (e.g., formant position for voiced sounds in speech, sharpness, and brightness) [4], [5]. But timbre is not only spectrum, as changes of amplitude over time, the so-called temporal envelope, also have strong perceptual effects [6], [7]. To identify the most salient timbre dimensions, statistical techniques such as multidimensional scaling have been used: perceptual differences between sound samples were collected and the underlying dimensionality of the timbre space inferred [8], [9]. These studies suggest a combination of spectral *and* temporal dimensions to explain the perceptual distance judgments, but the precise nature of these dimensions varies across studies and sound sets [10], [11]. Importantly, almost all timbre dimensions that have been proposed to date on the basis of psychophysical studies [12] are either purely spectral or purely temporal. The only spectro-temporal aspect of sound that has been considered in this context is related to the asynchrony of partials around the onset of a sound (8,9), but the salience of this spectro-temporal dimension was found to be weak and context-dependent [13].

Technological approaches, not concerned with biology nor human perception, have explored much richer feature representations that span both spectral, temporal, and spectro-temporal dimensions. The motivation for these engineering techniques is an accurate recognition of specific sounds or acoustic events in a variety of applications (e.g. automatic speech recognition; voice detection; music information retrieval; target tracking in multisensor networks and surveillance systems; medical diagnosis, etc.). Myriad spectral features have been proposed for audio content analysis, ranging from simple summary statistics of spectral shape (e.g. spectral amplitude, peak, centroid, flatness) to more elaborate descriptions of spectral information such as Mel-Frequency Cepstral Coefficients (MFCC) and Linear or Perceptual Predictive Coding (LPC or PLP) [14]–[16]. Such metrics have often been augmented with temporal information, which was found to improve the robustness of content identification [17], [18]. Common modeling of temporal dynamics also ranged from simple summary statistics such as onsets, attack time, velocity, acceleration and higher-order moments to more sophisticated statistical temporal modeling using Hidden Markov Models, Artificial Neural Networks, Adaptive Resonance Theory models, Liquid State Machine systems and Self-Organizing Maps [19], [20]. Overall, the choice of features was very dependent on the task at hand, the complexity of the dataset, and the desired performance level and robustness of the system.

Complementing perceptual and technological approaches, brain-imaging techniques have been used to explore the neural underpinnings of timbre perception. Correlates of musical timbre dimensions suggested by multidimensional scaling studies have been observed using event-related potentials [21]. Other studies have attempted to identify the neural substrates of natural sound recognition, by looking for brain areas that would be selective to specific sound categories, such as voice-specific regions in secondary cortical areas [22], [23] and other sound categories such as tools [24] or musical instruments [25]. A hierarchical model consistent with these findings has been proposed in which selectivity to different sound categories is refined as one climbs the processing chain [26]. An alternative, more distributed scheme has also been suggested [27], [28], which includes the contribution of low-level cues to the large perceptual differences between these high-level sound categories.

A common issue for the psychophysical, technological, and neurophysiological investigations of timbre is that the generality of the results is mitigated by the particular characteristics of the sound set used. For multi-dimensional scaling behavioral studies, by construction, the dimensions found will be the most salient within the sound set; but they may not capture other dimensions which could nevertheless be crucial for the recognition of sounds outside the set. For engineering studies, dimensions may be designed arbitrarily as long as they afford good performance in a specific task. For the imaging studies, there is no suggestion yet as to which low-level acoustic features may be used to construct the various selectivity for high-level categories while preserving invariance within a category. Furthermore, there is a major gap between these studies and what is known from electrophysiological recordings in animal models. Decades of work have established that auditory cortical responses display rich and complex spectro-temporal receptive fields, even within primary areas [29], [30]. This seems at odds with the limited set of spectral or temporal dimensions that are classically used to characterize timbre in perceptual studies.

To bridge this gap, we investigate how cortical processing of spectro-temporal modulations can subserve both sound source recognition of musical instruments and perceptual timbre judgments. Specifically, cortical receptive fields and computational models derived from them are shown to be suited to classify a sound source from its evoked neural activity, across a wide range of instruments, pitches and playing styles, and also to predict accurately human judgments of timbre similarities

## Results

### Cortical processing of complex musical sounds

**Figure 1:**
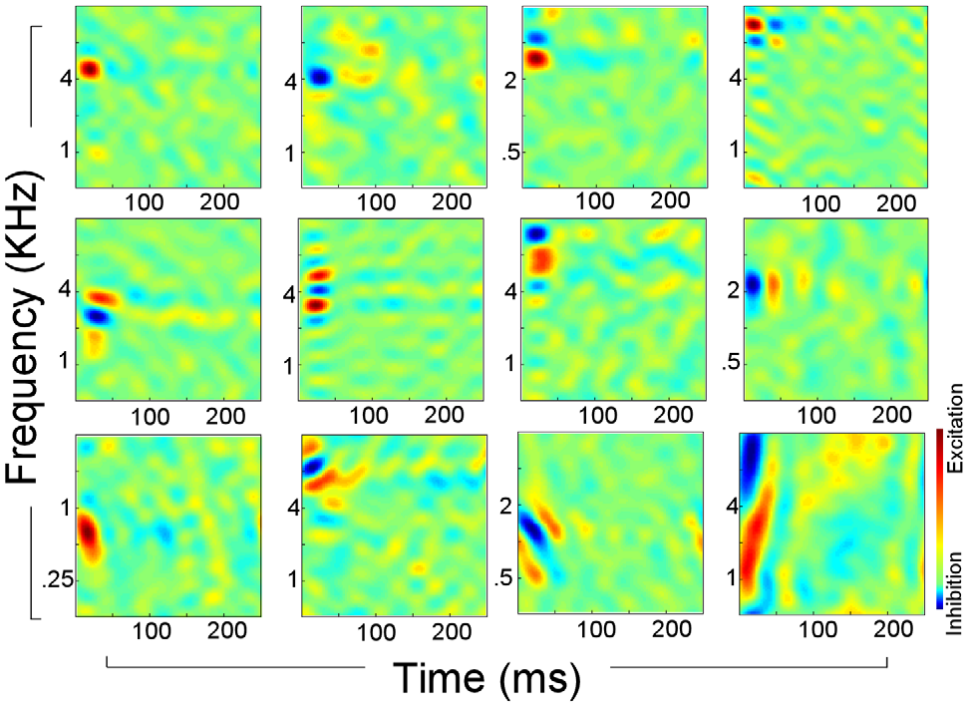
Neurophysiological receptive fields. Each panel shows the receptive field of 1 neuron with red indicating excitatory (preferred) responses, and blue indicating inhibitory (suppressed) responses. Examples vary from narrowly tuned neurons (top row) to broadly tuned ones (middle and bottom row). They also highlight variability in temporal dynamics and orientation (upward or downward sweeps).

Responses in primary auditory cortex (A1) exhibit rich selectivity that extends beyond the tonotopy observed in the auditory nerve. A1 neurons are not only tuned to the spectral energy at a given frequency, but also to the specifics of the local spectral shape such as its bandwidth [31], spectral symmetry [32], and temporal dynamics [33] (Figure 1). Put together, one can view the resulting representation of sound in A1 as a *multidimensional* mapping that spans at least three dimensions: (1) *Best frequencies* that span the entire auditory range; (2) *Spectral shapes* (including bandwidth and symmetry) that span a wide range from very broad (2–3 octaves) to narrowly tuned (<0.25 octaves); and (3) *Dynamics* that range from very slow to relatively fast (1–30 Hz).

This rich cortical mapping may reflect an elegant strategy for extracting acoustic cues that subserve the perception of various acoustic attributes (pitch, loudness, location, and timbre) as well as the recognition of complex sound objects, such as different musical instruments. This hypothesis was tested here by employing a database of spectro-temporal receptive fields (STRFs) recorded from 1110 single units in primary auditory cortex of 15 awake non-behaving ferrets. These receptive fields are *linear* descriptors of the selectivity of each cortical neuron to the spectral and temporal modulations evident in the cochlear “spectrogram-like” representation of complex acoustic signals that emerges in the auditory periphery. Such STRFs (with a variety of nonlinear refinements) have been shown to capture and predict well cortical responses to a variety of complex sounds like speech, music, and modulated noise [34]–[38].

**Figure 2:**
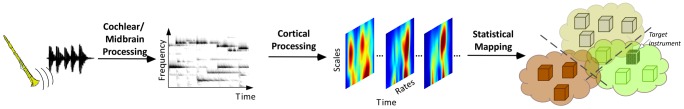
Schematic of the timbre recognition model. An acoustic waveform from a test instrument is processed through a model of cochlear and midbrain processing; yielding a time-frequency representation called auditory spectrogram. This later is further processed through the cortical processing stage through neurophysiological or model spectro-temporal receptive fields. Cortical responses of the target instrument are tested against boundaries of a statistical SVM timbre model in order to identify the instrument's identity.

To test the efficacy of STRFs in generating a representation of sound that can distinguish among a variety of complex categories, sounds from a large database of musical instruments were mapped onto cortical responses using the physiological STRFs described above. The time-frequency spectrogram for each note was convolved with each STRF in our neurophysiology database to yield a firing rate that is then integrated over time. This initial mapping was then reduced in dimensionality using singular value decomposition to a compact eigen-space; then augmented with a nonlinear statistical analysis using support vector machine (SVM) with Gaussian kernels [39] (see METHODS for details). Briefly, support vector machines are classifiers that learn to separate, in our specific case, the patterns of cortical responses induced by the different instruments. The use of Gaussian kernels is a standard technique that allows to map the data from its original space (where data may not be linearly separable) onto a new representational space that is linearly separable. Ultimately, the analysis constructed a set of hyperplanes that outline the boundaries between different instruments. The identity of a new sample was then defined based on its configuration in this expanded space relative to the set of learned hyperplanes (Figure 2).

Based on the configuration above and a 10% cross-validation technique, the model trained using the physiological cortical receptive fields achieved a classification accuracy of **87.22%±0.81** (the number following the mean accuracy represents standard deviation, see Table 1). Remarkably, this result was obtained with a large database of 11 instruments playing between 30 and 90 different pitches with 3 to 19 playing styles (depending on the instrument), 3 style dynamics (mezzo, forte and piano), and 3 manufacturers for each instrument (an average of 1980 notes/instrument). This high classification accuracy was a strong indicator that neural processing at the level of primary auditory cortex could not only provide a basis for distinguishing between different instruments, but also had a robust invariant representation of instruments over a wide range of pitches and playing styles.

**Table 1:** Classification performance for the different models.

	Mean	STD
Auditory Spectrum (Gaussian kernel SVM)	79.1%	0.7%
Neurophysiological STRFs (Gaussian kernel SVM)	87.2%	0.8%
Full Cortical Model (Linear SVM)	96.2%	0.5%
Full Cortical Model (Gaussian kernel SVM)	98.7%	0.2%

The middle column indicates the mean of the accuracy scores for the 10 fold cross validation experiment and the right column indicates their standard deviation. Models differ either in their feature set (e.g. full cortical model versus auditory spectrogram) or in the classifier used (linear SVM versus Gaussian kernel SVM).

### The cortical model

Despite the encouraging results obtained using cortical receptive fields, the classification based on neurophysiological recordings was hampered by various shortcomings including recording noise and other experimental constraints. Also, the limited selection of receptive fields (being from ferrets) tended to under-represent parameter ranges relevant to humans such as lower frequencies, narrow bandwidths (limited to a maximum resolution of 1.2 octaves), and coarse sampling of STRF dynamics.

To circumvent these biases, we employed a model that mimics the basic transformations along the auditory pathway up to the level of A1. Effectively, the model mapped the one-dimensional acoustic waveform onto a multidimensional feature space. Importantly, the model allowed us to sample the cortical space more uniformly than physiological data available to us, in line with findings in the literature [29], [30], [40].

**Figure 3:**
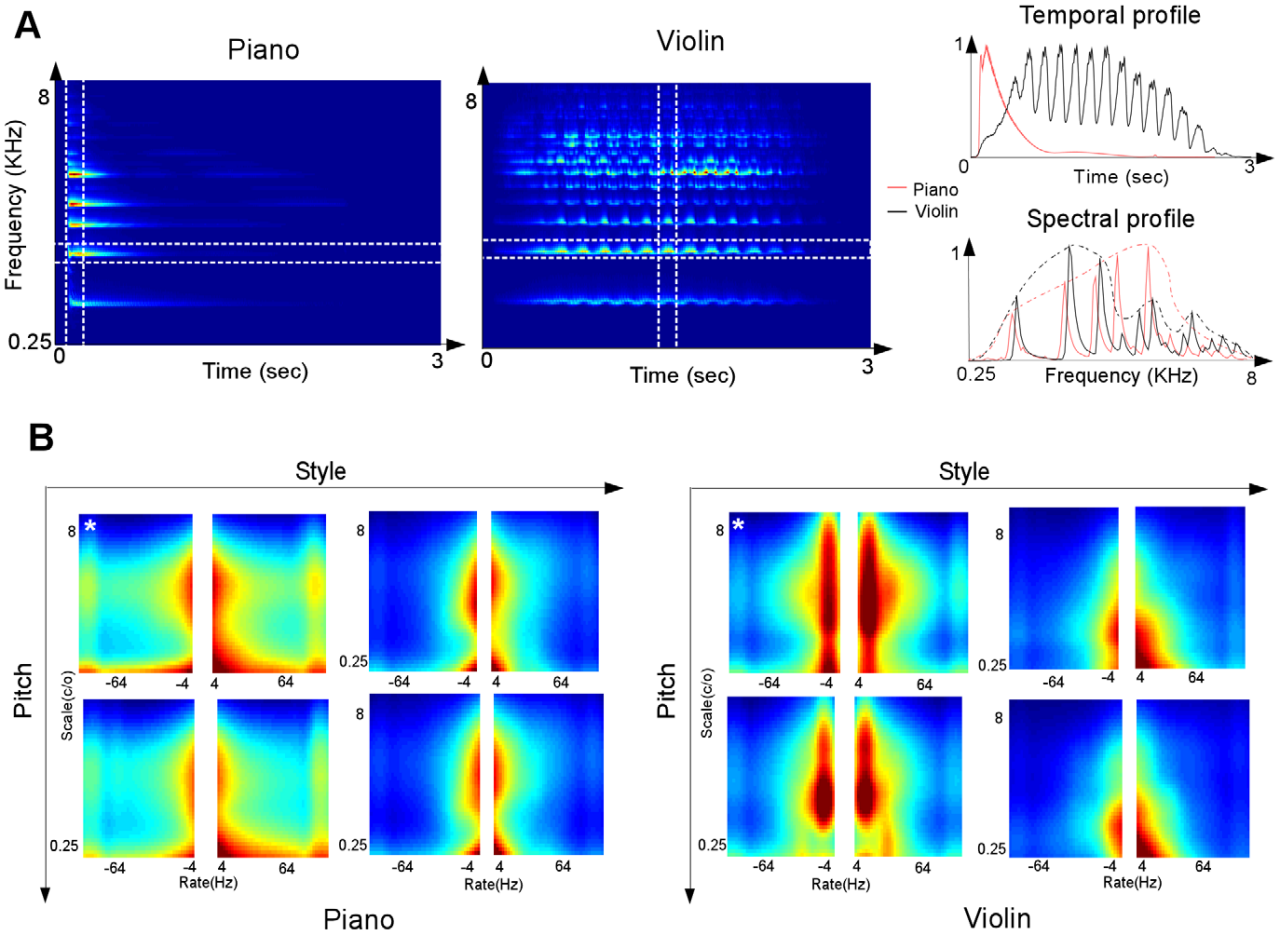
Spectro-temporal modulation profiles highlighting timbre differences between piano and violin notes. (**A**) The plot shows the time-frequency auditory spectrogram of piano and violin notes. The temporal and spectral slices shown on the right are marked. (**B**) The plots show magnitude cortical responses of four piano notes (left panels), played in normal (left) and Staccato (right) at F4 (top) and F#4 (bottom); and four violin notes (right panels), played in normal (left) and Pizzicatto (right) also at pitch F4(top) and F#4 (bottom). The white asterisks (upper leftmost notes in each quadruplet) indicate the notes shown in part (**A**) of this figure.

The model operates by first mapping the acoustic signal into an auditory spectrogram. This initial transformation highlights the time varying spectral energies of different instruments which is at the core of most acoustic correlates and machine learning analyses of musical timbre [5], [11], [13], [41], [42]. For instance, temporal features in a musical note include fast dynamics that reflect the quality of the sound (scratchy, whispered, or purely voiced), as well as slower modulations that carry nuances of musical timbre such as attack and decay times, subtle fluctuations of pitch (vibrato) or amplitude (shimmer). Some of these characteristics can be readily seen in the auditory spectrograms, but many are only implicitly represented. For example, Figure 3A contrasts the auditory spectrogram of a piano vs. violin note. For violin, the temporal cross-section reflects the soft onset and sustained nature of bowing and typical vibrato fluctuations; the spectral slice captures the harmonic structure of the musical note with the overall envelope reflecting the resonances of the violin body. By contrast, the temporal and spectral modulations of a piano (playing the same note) are quite different. Temporally, the onset of piano rises and falls much faster, and its spectral envelope is much smoother.

The cortical stage of the auditory model further analyzes the spectral and temporal modulations of the spectrogram along multiple spectral and temporal resolutions. The model projects the auditory spectrogram onto a 4-dimensional space, representing time, tonotopic frequency, spectral modulations (or scales) and temporal modulations (or rates). The four dimensions of the cortical output can be interpreted in various ways. In one view, the cortical model output is a parallel repeated representation of the auditory spectrogram viewed at different resolutions. A different view is one of a bank of spectral and temporal modulation filters with different tuning (from narrowband to broadband spectrally, and slow to fast modulations temporally). In such view, the cortical representation is a display of spectro-temporal modulations of each channel as they evolve over time. Ultimately each filter acts as a model cortical neuron whose output reflects the tuning of that neuronal site. The model employed here had 30,976 filters (128freq×22 rates×11 scales), hence allowing us to obtain a full uniform coverage of the cortical space and bypassing the limitations of neurophysiological data. Note that we are not suggesting that ∼30 K neurons are needed for timbre classification, as the feature space is reduced in further stages of the model (see below). We have not performed an analysis of the number of neurons needed for such task. Nonetheless, a large and uniform sampling of the space seemed desirable.

By collapsing the cortical display over frequency and averaging over time, one would obtain a two-dimensional display that preserves the “global” distribution of modulations over the remaining two dimensions of scale and rates. This “scale-rate” view is shown in Figure 3B for the same piano and violin notes in Figure 3A as well as others. Each instrument here is played at two distinct pitches with two different playing styles. The panels provide estimates of the overall distribution of spectro-temporal modulation of each sound. The left panel highlights the fact that the violin vibrato concentrates its peak energy near 6 Hz (across all pitches and styles); which matches the speed of pulsating pitch change caused by the rhythmic rate of 6 pulses per second chosen for the vibrato of this violin note. By contrast, the rapid onset of piano distributes its energy across a wider range of temporal modulations. Similarly, the unique pattern of peaks and valleys in spectral envelopes of each instrument produces a broad distribution along the spectral modulation axis, with the violin's sharper spectral peaks activating higher spectral modulations while the piano's smoother profile activates broad bandwidths. Each instrument, therefore, produces a correspondingly unique spectro-temporal activation pattern that could potentially be used to recognize it or distinguish it from others.

### Musical timbre classification

Several computational models were compared in the same classification task analysis of the database of musical instruments as described earlier with real neurophysiological data. Results comparing all models are summarized in Table 1. For what we refer to as the full model, we used the 4-D cortical model. The analysis started with a linear mapping through the model receptive fields, followed by dimensionality reduction and statistical classification using support vector machines with re-optimized Gaussian kernels (see Methods). Tests used a 10% cross-validation method. The cortical model yielded an excellent classification accuracy of **98.7%±0.2**.

We also explored the use of linear support vector machine, by bypassing the use of the Gaussian kernel. We performed a classification of instruments using the cortical responses obtained from the model receptive fields and a linear SVM. After optimization of the decision boundaries, we obtained an accuracy of **96.2%±0.5**. This result supports our initial assessment that the cortical space does indeed capture most of the subtleties that are unique to a common instrument but distinct between different classes. It is mostly the richness of the representation that underlies the classification performance: only a small improvement in accuracy is observed by adding the non-linear warping in the full model.

**Figure 4:**
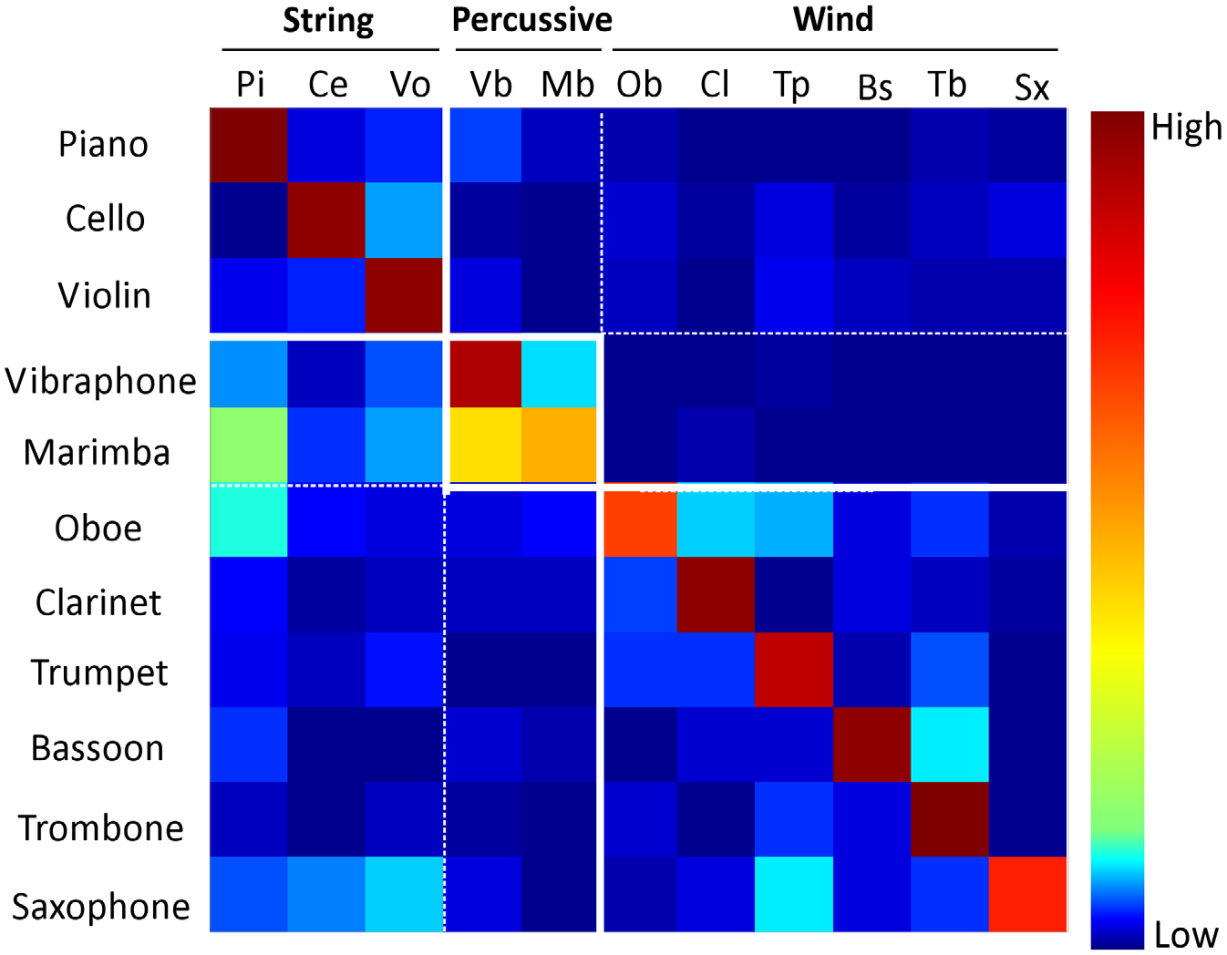
The confusion matrix for instrument classification using the auditory spectrum. Each row sums to 100% classification (with red representing high values and blue low values). Rows represent instruments to be identified and columns are instrument classes. Off diagonal values that are non-dark blue represent errors in classification. The overall accuracy from this confusion matrix is 79.1%±0.7.

In order to better understand the contribution of the cortical analysis beyond the time-frequency representation, we explored reduced versions of the full model. First we performed the timbre classification task using the auditory spectrogram as input. The feature spectra were obtained by processing the time waveform of each note through the cochlear-like filterbank front-end and averaging the auditory spectrograms over time, yielding a one-dimensional spectral profile for each note. These were then processed through the same statistical SVM model, with Gaussian functions optimized for this new representation using the exact same methods as used for cortical features. The classification accuracy for the spectral slices with SVM optimization attained a good but limited accuracy of **79.1%±0.7**. It is expected that a purely spectral model would not be able to classify all instruments. Whereas basic instrument classes differing by their physical characteristics (wind, percussion, strings) may have the potential to produce different spectral shapes, preserved in the spectral vector, more subtle differences in the temporal domain should prove difficult to recognize on this basis (see Figure 4). We shall revisit this issue of contribution and interactions between spectral and temporal features later (see Control Experiments section).

**Figure 5:**
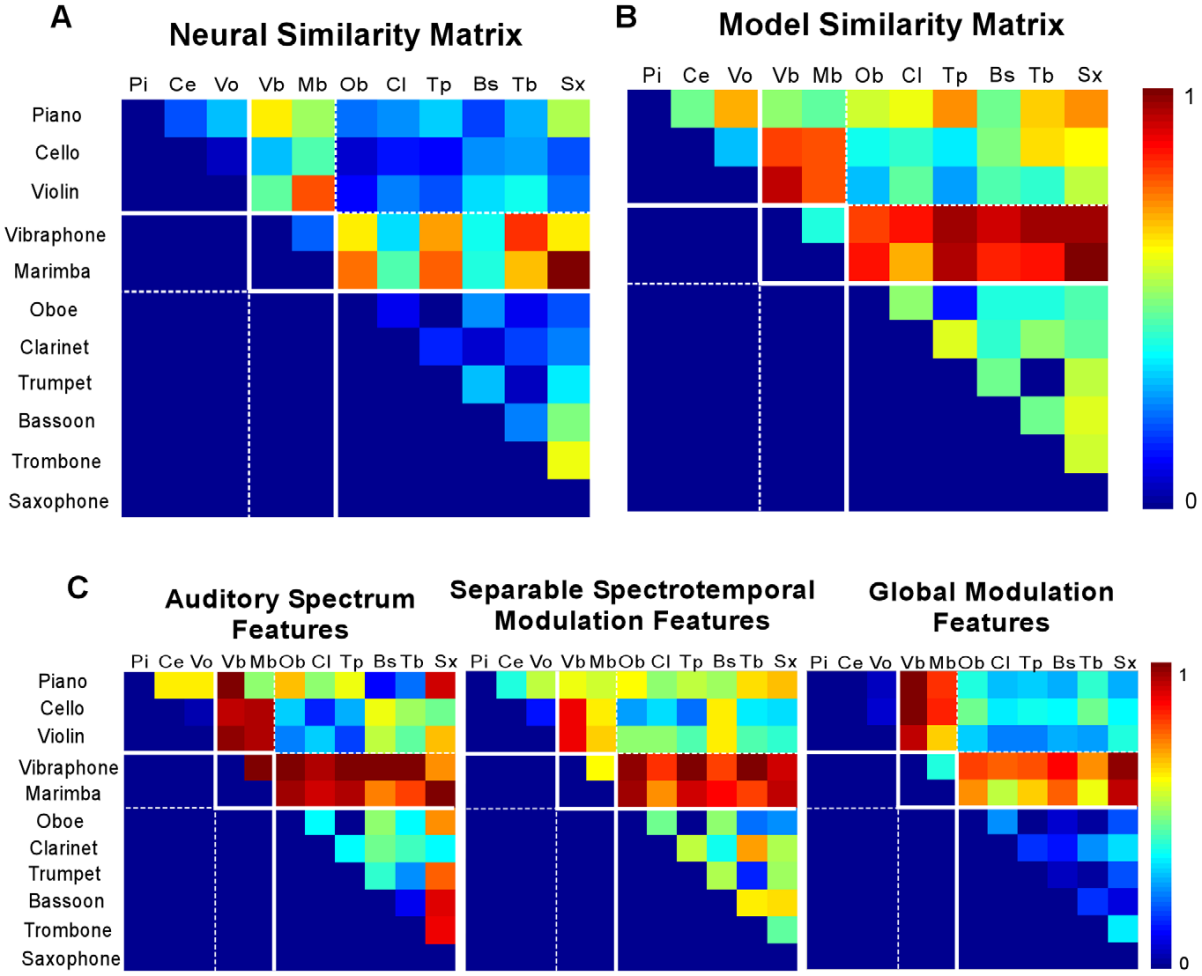
The average KL divergence between support vectors of instruments belonging to different broad classes. Each panel depicts the values of the 3 dimensional average distances between pairs of instruments of a given couple of classes: (**A**) wind vs. percussion; (**B**) string vs. percussion; (**C**) wind vs. string. The 3 dimensional vectors are displayed along eigenrates (x-axis), eigenscales (y-axis) and eigenfrequency (across small subpanels). Red indicates high values of KL divergence and blue indicates low values.

**Figure 6:**
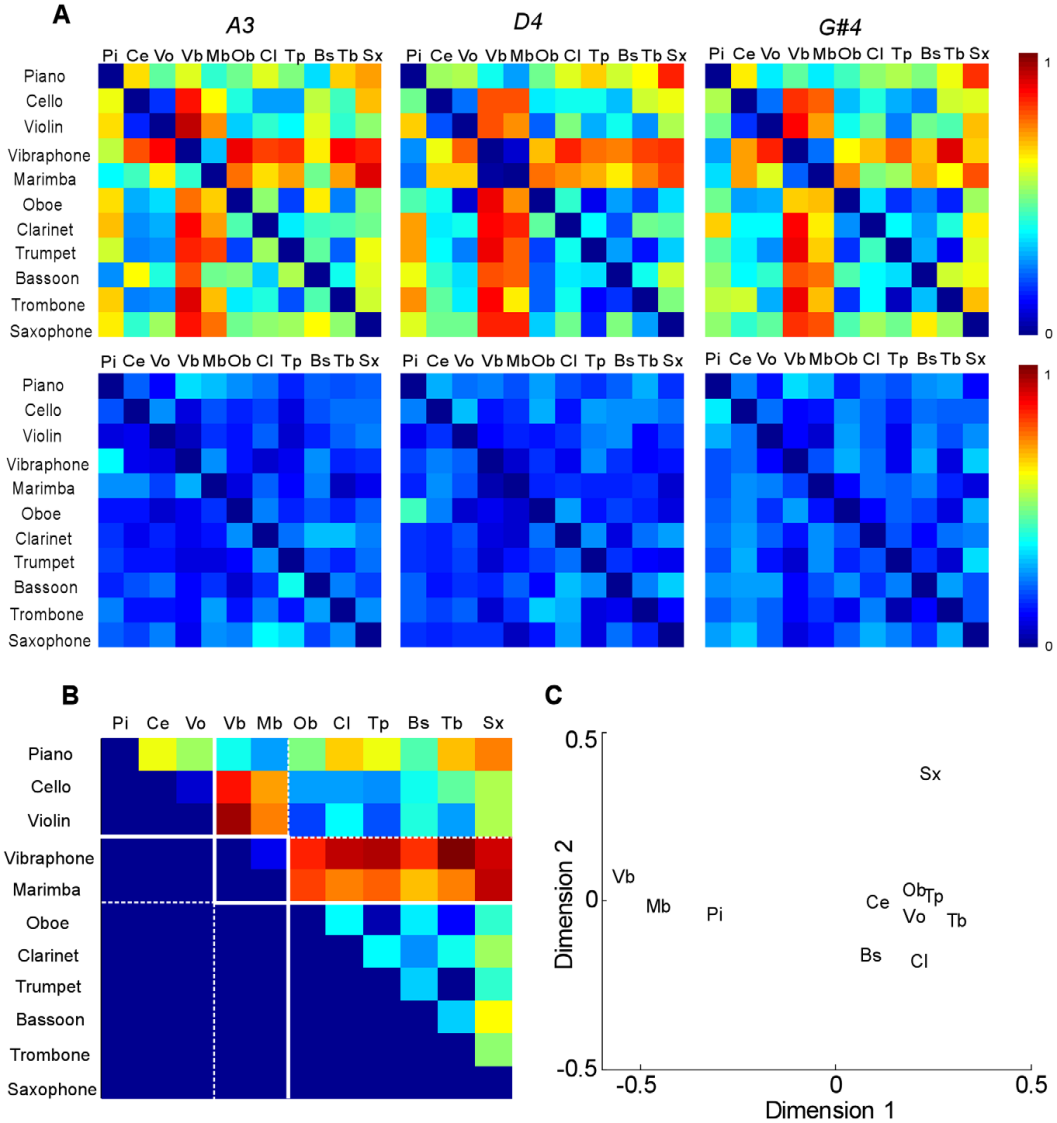
Human listener's judgment of musical timbre similarity. (**A**) The mean (top row) and standard deviation (bottom row) of the listeners' responses show the similarity between every pair of instruments for three notes A3, D4 and G#4. Red (values close to 1) indicates high dissimilarity and blue (values close to 0) indicates similarity. (**B**) Timbre similarity is averaged across subjects, musical notes and upper and lower half-matrices, and used for validation of the physiological and computational model. (**C**) Multidimensional scaling (MDS) applied to the human similarity matrix projected over 2 dimensions (shown to correlate with attack time and spectral centroid).

We performed a post-hoc analysis of the decision space based on cortical features in an attempt to get a better understanding of the configuration of the decision hyperplanes between different instrument classes. The analysis treated the support vectors (i.e. samples of each instrument that fall right on the boundary that distinguishes it from another instrument) for each instrument as samples from an underlying high-dimensional probability density function. Then, a measure of similarity between pairs of probability functions (symmetric Kullback–Leibler (KL) divergence [43]) was employed to provide a sense of distance between each instrument pair in the decision space. Because of the size and variability in the timbre decision space, we pooled the comparisons by instrument class (winds, strings and percussions). We also focused our analysis on the reduced dimensions of the cortical space; called ‘eigen’-rate, ‘eigen’-scale and ‘eigen’-frequencies; obtained by projecting the equivalent dimensions in the cortical tensor (rate, scale and frequency, respectively) into a reduced dimensional space using singular-value decomposition (see METHODS). The analysis revealed a number of observations (see Figure 5). For instance, wind and percussion classes were the most different (occupy distant regions in the decision space), followed by strings and percussions then strings and winds (average KL distances were 0.58, 0.41, 0.35, respectively). This observation was consistent with the subjective judgments of human listeners presented next (see off-diagonal entries in Figure 6B). All 3 pair comparisons were statistically significantly different from each other (Wilcoxon ranksum test, p<10^−5^ for all 3 pairs). Secondly, the analysis revealed that the 2 first ‘eigen’-rates captured most of the difference between the instrument classes (statistical significance in comparing the first 2 eigenrates with the others; Wilcoxon ranksum test, p = 0.0046). In contrast, *all* ‘eigen’-scales were variable across classes (Kruskal-Wallis test, p = 0.9185 indicating that all ‘eigen’-scales contributed equally in distinguishing the broad classes). A similar analysis indicated that the first four ‘eigen’-frequencies were also significantly different from the remaining features (Wilcoxon ranksum test, p<10^−5^). One way to interpret these observations is that the first two principal orientations along the rate axis captured most of the differences that distinguish winds, strings and percussions. This seems consistent with the large differences in temporal envelope shape for these instruments classes, which can be represented by a few rates. By contrast, the scale dimension (which captures mostly spectral shape, symmetry and bandwidth) was required in its entirety to draw a boundary between these classes, suggesting that unlike the coarser temporal characteristics, differentiating among instruments entails detailed spectral distinctions of a subtle nature.

### Comparison with standard classification algorithms

Spectral features have been extensively used for tasks of musical timbre classification of isolated notes, solo performances or even multi-instrument recordings. Features such as Cepstral Coefficients or Linear Prediction of the spectrum resonances yielded performance in the range of 77% to 90% when applied to databases similar to the one used in the present study [44]–[46].

There is wide agreement in the literature that inclusion of simple temporal features, such as zero-crossing rate, or more complex ones such as trajectory estimation of spectral envelopes, is often desirable and results in improvement of the system performance. Tests on the RWC database with both spectral and temporal features reported an accuracy of 79.7% using 19 instruments [47] or 94.9% using 5 instruments [42]. Tests of spectrotemporal features on other music databases has often yielded a range of performances between 70–95% [48]–[51].

Whereas a detailed comparisons with our results is beyond the scope of this paper, we can still note that, if anything, the recognition rates we report for the full auditory model are generally in the range or above those reported by state-of-the-art signal processing techniques.

### Psychophysics timbre judgments

Given the ability of the cortical model to capture the diversity of musical timbre across a wide range of instruments in a classification task, we next explored how well the cortical representation (from both real and model neurons) does in capturing human perceptual judgments of distance in the musical timbre space. To this end, we used human judgments of musical timbre distances using a psychoacoustic comparison paradigm.

Human listeners were asked to rate the similarity between musical instruments. We used three different notes (A3, D4 and G#4) in three different experiments. Similarity matrices for all three notes yielded reasonably balanced average ratings across subjects, instrument pair order (e.g. piano/violin vs. violin/piano) and pitches, in agreement with other studies [52] (Figure 6A). Therefore, we combined the matrices across notes and listeners into an upper half matrix shown in Figure 6B, and used it for all subsequent analyses. For comparison with previous studies, we also ran a multidimensional scaling (MDS) analysis [53] on this average timbre similarity rating and confirmed that the general configuration of the perceptual space was consistent with previous studies (Figure 6C) [8]. Also for comparison, we tested acoustical dimensions suggested in those studies. The first dimension of our space correlated strongly with the logarithm of attack-time (Pearson's correlation coefficient: ρ = 0.97, p<10^−3^), and the second dimension correlated reasonably well with the center of mass of the auditory spectrogram, also known as spectral centroid (Pearson's correlation coefficient: ρ = 0.62, p = 0.04).

### Human vs. model timbre judgments

**Figure 7:**
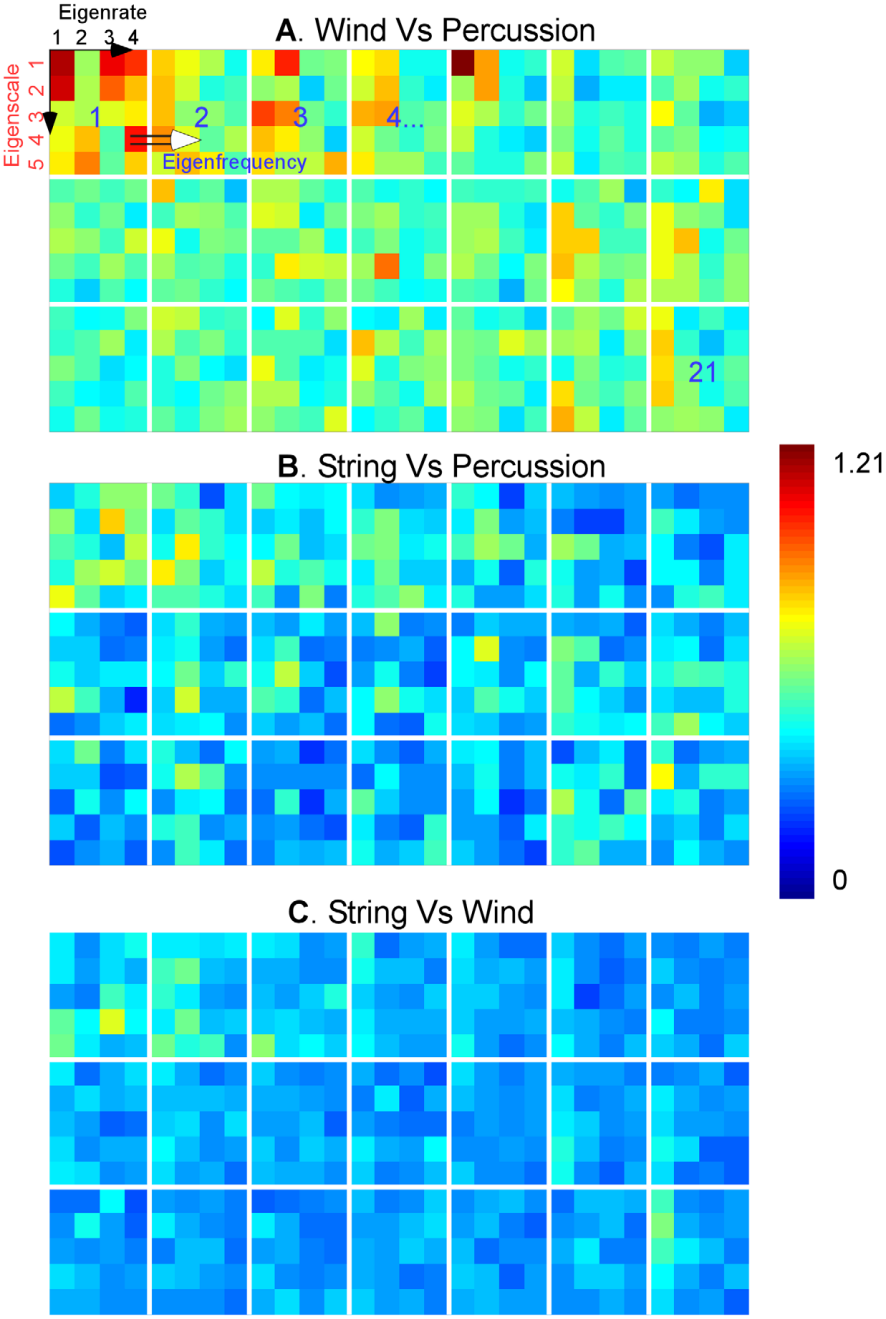
Model musical timbre similarity. Instrument similarity matrices based kernel optimization technique of the (**A**) neurophysiological receptive field and (**B**) cortical model receptive fields. (**C**) Control experiments using the auditory spectral features (left), separable spectro-temporal modulation feature (middle), and global modulation features [separable spectral and temporal modulations integrated across time and frequency] (right). Red depicts high dissimilarity. All the matrices show only the upper half-matrix with the diagonal not shown.

The perceptual results obtained above, reflecting subjective timbre distances between different instruments, summarizes an elaborate set of judgments that potentially reveal other facets of timbre perception than the listeners' ability to recognize instruments. We then explored whether the cortical representation could account for these judgments. Specifically, we asked whether the cortical analysis maps musical notes onto a feature space where instruments like violin and cello are distinct, yet closer to each other than a violin and a trumpet. We used the same 11 instruments and 3 pitches (A3, D4 and G#4) employed in the psychoacoustics experiment above and mapped them onto a cortical representation using both neurophysiological and model STRFs. Each note was then vectorized into a feature data-point and mapped via Gaussian kernels. These kernels are similar to the radial basis functions used in the previous section, and aimed at mapping the data from its original cortical space to a linearly separable space. Unlike the generic SVM used in the classification of musical timbre, the kernel parameters here were optimized based on the human scores following a similarity-based objective function. The task here was not merely to classify instruments into distinct classes, but rather to map the cortical features according to a complex set of rules. Using this learnt mapping, a confusion matrix was constructed based on the instrument distances, which was then compared with the human confusion matrix using a Pearson's correlation metric. We performed a comparison with the physiological as well as model STRFs. The simulated confusion matrices are shown in Figure 7A–B.

The success or otherwise of the different models was estimated by correlating the human dissimilarity matrix to that generated by the model. No attempt was made at producing MDS analyses of the model output, as meaningfully comparing MDS spaces is not a trivial problem [52]. Physiological STRFs yielded a correlation coefficient of **0.73**, while model STRFs yielded a correlation of **0.94** (Table 2).

**Table 2:** Correlation coefficients for different feature sets.

	L2 on features	L2 on reduced features	Gaussian kernels on reduced features
Fourier-based Spectrum	-	-	0.69
Auditory Spectrum	0.473	-	0.739
Global Spectro-temporal Modulations	0.509	-	0.701
Separable Spectro-temporal Modulations	0.561	0.561	0.830
Full cortical Model	0.611	0.607	0.944
Neurophysiological STRFs	-	-	0.73

Each row represents the correlation coefficient between the model and human similarity matrix using a direct Euclidian distance the specific feature itself (left); on a reduced dimension of the features (middle column) or using the Gaussian kernel distance (right column). Auditory Spectrum: time-average of the cochlear filterbank; Global Spectro-temporal Modulations: model STRFs averaged in time and frequency; Separable Spectro-temporal modulations: model STRFs averaged separately in rate and scale, and then in time; Full cortical model: STRFs averaged in time; Neurophysiological STRFs: as the full cortical model, but with STRFs collected in primary auditory cortex of ferrets.

### Control experiments

In order to disentangle the contribution of the “input” cortical features versus the “back-end” machine learning in capturing human behavioral data, we recomputed confusion matrices using alternative representations such as the auditory spectrogram and various marginals of the cortical distributions. In all these control experiments, the Gaussian kernels were re-optimized separately to fit the data representation being explored.

We first investigated the performance using auditory spectrum features with optimized Gaussian kernels. The spectrogram representation yielded a similarity matrix that captures the main trends in human distance judgments, with a correlation coefficient of **0.74** (Figure 7C, leftmost panel). Similar experiments using a traditional spectrum (based on Fourier analysis of the signal) yield a correlation of **0.69**.

Next, we examined the effectiveness of the model cortical features by reducing them to various marginal versions with fewer dimensions as follows. First, we performed an analysis of the spectral and temporal modulations as a *separable* cascade of two operations. Specifically, we analyzed the spectral profile of the auditory spectrogram (scales) *independently* from the temporal dynamics (rates) and stack the two resulting feature vectors together. This analysis differed from the full cortical analysis that assumes an inseparable analysis of spectro-temporal features. An inseparable function is one that cannot be factorized into a function of time and a function of frequency; i.e. a matrix of rank greater than 1 (see Methods). By construction, a separable function consists of temporal cross sections that are scaled versions of the same essential temporal function. A consequence of such constraint is that a separable function cannot capture orientation in time-frequency space (e.g. FM sweeps). In contrast, the full cortical analysis estimates modulations along *both* time and frequency axes in addition to an integrated view of the two axes including orientation information The analysis based on the separable model achieved a correlation coefficient of **0.83** (Table 2).

Second, we further reduced the separable spectro-temporal space by analyzing the modulation content along both time and frequency without maintaining the distribution along the tonotopic axis. This was achieved by simply integrating the modulation features along the spectral axis thus exploring the global characteristic of modulation regardless of tonotopy (Figure 7C, rightmost panel). This representation is somewhat akin to what would result from a 2-dimensional Fourier analysis of the auditory spectrogram. This experiment yielded a correlation coefficient of **0.70** (Table 2), supporting the value of an explicit tonotopic axis in capturing subtle difference between instruments.

**Figure 8:**
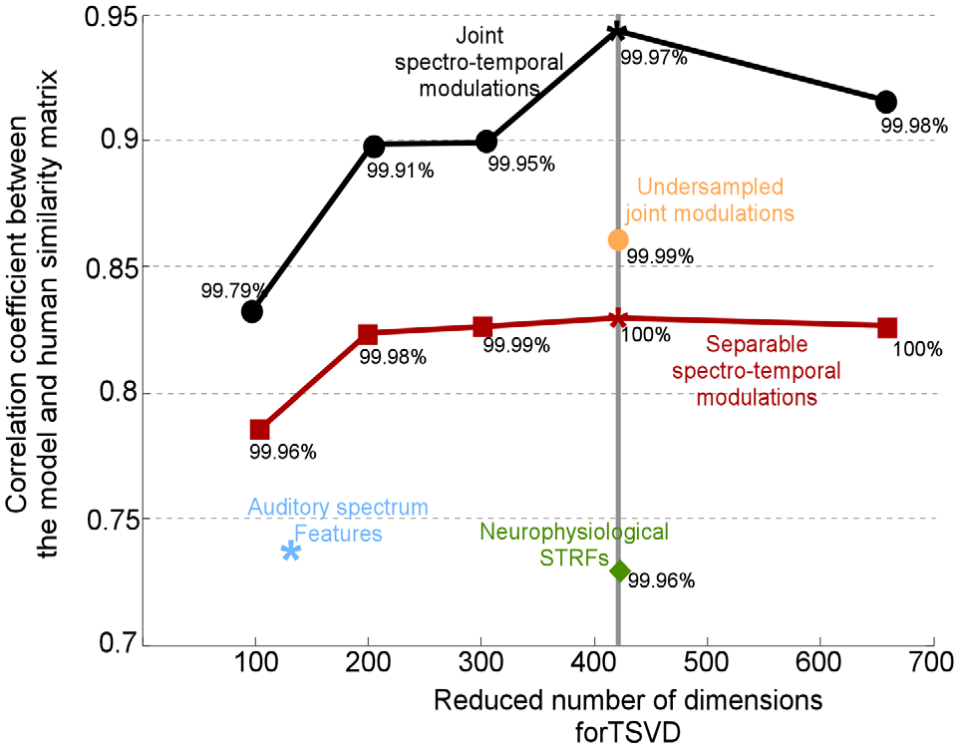
Correlation between human and model similarity matrices as a function of reduced feature dimensionality. In each simulation, a cortical representation is projected onto a lower dimensional space before passing it onto the SVM classifier. Each projection maintains a given percentage of the variability in the original data (shown adjacent to each correlation data point). We contrast performance using full cortical model (black curve) vs. separable spectro-temporal modulation model (red curve). The empirical optimal performance is achieved around 420 dimensions; which are the parameters reported in the main text in table 2.

Next, we addressed the concern that the mere number of features included in the full cortical model enough to explain the observed performance. We therefore undersampled the full cortical model by employing only 6 scale filters; 10 rate filters and 64 frequency filters by coarsely sampling the range of spectro-temporal modulations. This mapping resulted in a total number of dimensions of 3840; to be comparable to the 4224 dimensions obtained from the separable model. We then performed the dimensionality reduction to 420 dimensions, similar to that used for the separable analysis discussed above. The correlation obtained was **0.86**; which is better than that of the separable spectro-temporal model (see Figure 8). This result supports our main claim that the *coverage* provided by the cortical space allows extracting specific details in the musical notes that highlight information about the physical properties of each instrument; hence enabling classification and recognition.

Finally, we examined the value of the kernel-learning compared to using a simple Euclidian L2 distance at various stages of the model (e.g. peripheral model, cortical stage, reduced cortical model using tensor singular value decomposition). Table 2 summarizes the results of this analysis along various stages of the model shown in Figure 2. The analysis revealed that the kernel-based mapping does provide noticeable improvement to the predictive power of the model but cannot –by itself– explain the results since the same technique applied directly on the spectrum only yielded a correlation of **0.74**.

## Discussion

This study demonstrates that perception of musical timbre could be effectively based on neural activations patterns that sounds evoke at the level of primary auditory cortex. Using neurophysiological recordings in mammalian auditory cortex as well as a simplified model of cortical processing, it is possible to accurately replicate human perceptual similarity judgments and classification performance among sounds from a large number of musical instruments. Of course, showing that the information is available at the level of primary auditory cortex does not imply that all neural correlates of sound identification will be found at this level. Nevertheless, it suggests that the spectro-temporal transforms as observed at this stage are critical for timbre perception. Moreover, our analysis highlights the ability of the cortical mapping to capture timbre properties of musical notes and instrument-specific characteristics regardless of pitch and playing style. Unlike static or reduced views of timbre that emphasize three or four parameters extracted from the acoustic waveform, the cortical analysis provides a dynamic view of the spectro-temporal modulations in the signal as they vary over time. A close examination of the contribution of different auditory features and processing stages to the timbre percepts highlights three key points.

First, neither the traditional spectrum nor its variants (e.g. average auditory spectrum [54]) are well-suited to account for timbre perception in full. According to our simulations, these representations encode the relevant spectral and temporal acoustic features too implicitly to lend themselves for exploitation by classifiers and other machine learning techniques. In some sense, this conclusion is expected given the multidimensional nature of the timbre percept compared to the dense two-dimensional spectrogram; and is in agreement with other findings from the literature [19].

Second, when considering more elaborate spectro-temporal cortical representations, it appears that the *full* representation accounts best for human performance. The match worsens if instead *marginals* are used by collapsing the cortical representation onto one or more dimensions to extract the purely spectral or temporal axes or scale-rate map (Figure 3, Tables 1 and 2). This is the case even if all dimensions are used separately, suggesting that there are *joint* spectro-temporal features that are key to a full accounting of timbre. While the role of both purely spectral and temporal cues in musical timbre is quite established [12], our analysis emphasizes the crucial contribution of a joint spectro-temporal representation. For example, FM modulations typical of vibrato in string instruments are joint features that cannot be easily captured by the marginal spectral or temporal representations. Interestingly, acoustical analyses and fMRI data in monkeys suggest that the spectro-temporal processing scheme used here may be able to differentiate between broad sound categories (such as monkey calls vs. bird calls vs. human voice), with corresponding neural correlates when listening to those sounds [55].

Third, a nonlinear decision boundary in the SVM classifier is essential to attain the highest possible match between the cortical representation and human perception. Linear metrics such as L2 are less optimal, indicating that the linear cortical representation may not be sufficiently versatile to capture the nuances of various timbres. The inadequacy of the *linear* cortical mapping has previously been described when analyzing neural responses to complex sounds such as speech at the level of auditory cortex [35], [36], [38]. In these cases, it is necessary to postulate the existence of nonlinearities such as divisive normalization or synaptic depression that follows a linear spectro-temporal analysis so as to account fully for the observed responses. In the current study, the exact nature of the nonlinearity remains unclear as it is implicitly subsumed in the Gaussian kernels and subsequent decisions.

In summary, this study leads to the general conclusion that timbre percepts can be effectively explained by the joint spectro-temporal analysis performed at the level of mammalian auditory cortex. However, unlike the small number of spectral or temporal dimensions that have been traditionally considered in the timbre literature, we cannot highlight a simple set of neural dimensions subserving timbre perception. Instead, the model suggests that subtle perceptual distinctions exhibited by human listeners are based on ‘opportunistic’ acoustic dimensions [56] that are selected and enhanced, when required, on the rich baseline provided by the cortical spectro-temporal representation.

## Methods

### Ethics statement

All behavioral recordings of timbre similarity judgments with human listeners were approved by the local ethics committee of the Université Paris Descartes. All procedures for recordings of single unit neural activity in ferrets were in accordance with the Institutional Animal Care and Use Committee at the University of Maryland, College Park and the Guidelines of the National Institutes of Health for use of animals in biomedical research.

### Psychoacoustics

#### Stimuli

Recordings of single musical notes were extracted from the RWC Music Database [57], using the notes designated “medium-volume” and “staccato”, with pitches of A3, D4, and G#4. The set used for the experiments comprised 13 sound sources: Piano, Vibraphone, Marimba, Cello, Violin, Oboe, Clarinet, Trumpet, Bassoon, Trombone, Saxophone, male singer singing the vowel /a/, male singer singing the vowel /i/. Each note was edited into a separate sound file, truncated to 250 ms duration with 50 ms raised cosine offset ramp (the onset was preserved), and normalized in RMS power. More details on the sound set can be found in [56]. The analyses presented in the current study exclude the results from the 2 vowels, as only musical instruments were considered in the model classification experiments.

#### Participants and apparatus

A total of twenty listeners participated in the study (14 totally naïve participants, 6 participants experienced in psychoacoustics experiment but naïve to the aim of the present study; mean age: 28 y; 10 female). They had no self-reported history of hearing problems. All twenty subjects performed the test with the D4 pitch. Only six took part in the remaining tests with notes A3 and G#4. Results from the 6 subjects tested on all 3 notes are reported here, even though we checked that including all subjects would not change our conclusions. Stimuli were played through an RME Fireface sound-card at a 16-bit resolution and a 44.1 kHz sample-rate. They were presented to both ears simultaneously through Sennheiser HD 250 Linear II headphones. Presentation level was 65 dB(A). Listeners were tested individually in a double-walled IAC sound booth.

#### Procedure

Subjective similarity ratings were collected. For a given trial, two sounds were played with a 500 ms silent interval. Participants had to indicate how similar they perceived the sounds to be. Responses were collected by means of a graphical interface with a continuous slider representing the perceptual similarity scale. The starting position of the slider was randomized for each trial. Participants could repeat the sound pair as often as needed before recording their rating. In an experimental block, each sound was compared to all others (with both orders of presentations) but not with itself. This gave a total of 156 trials per block, presented in random order. Before collecting the experimental data, participants could hear the whole sound set three times. A few practice trials were also provided until participants reported having understood the task and instructions. A single pitch was used for all instruments in each block; the three types of blocks (pitch A3, D4, or G#4) were run in counterbalanced order across participants. Two blocks per pitch were run for each participants, and only the second block was retained for the analysis.

#### Multidimensional scaling (MDS) and acoustical correlates

To compare the results with previous studies, we ran an MDS analysis on the dissimilarity matrix obtained from human judgments. A standard non-metric MDS was performed (Matlab, the MathWorks). Stress values were generally small, with a knee-point for the solution at two dimensions (0.081, Kruskal normalized stress1). We also computed acoustical descriptors corresponding to the classic timbre dimensions. Attack time was computed by taking the logarithm of the time taken to go from −40 dB to −12 dB relative to the maximum waveform amplitude. Spectral centroid was computed by running the stimuli in an auditory filterbank, compressing the energy in each channel (exponent: 0.3), and taking the center of mass of the resulting spectral distribution.

### Auditory model

The cortical model is comprised of two main stages: an early stage mimicking peripheral processing up to the level of the midbrain, and a central stage capturing processing in primary auditory cortex (A1). Full details about the model can be found in [54], [58]; but are described briefly here.

The processing of the acoustic signal in the cochlea is modeled as a bank of 128 constant-Q asymmetric bandpass filters equally spaced on the logarithmic frequency scale spanning 5.3 octaves. The cochlear output is then transduced into inner hair cells potentials via a high pass and low pass operation. The resulting auditory nerve signals undergo further spectral sharpening via a lateral inhibitory network. Finally, a midbrain model resulting in additional loss in phase locking is performed using short term integration with time constant 4 ms resulting in a time frequency representation called as the auditory spectrogram.

The central stage further analyzes the spectro-temporal content of the auditory spectrogram using a bank of modulation selective filters centered at each frequency along the tonotopic axis, modeling neurophysiological receptive fields. This step corresponds to a 2D affine wavelet transform, with a spectro-temporal mother wavelet, define as Gabor-shaped in frequency and exponential in time. Each filter is tuned (Q = 1) to a specific rate (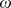 in Hz) of temporal modulations and a specific scale of spectral modulations (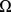 in cycles/octave), and a directional orientation (+ for upward and − for downward).

For input spectrogram 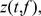 the response of each STRF in the model is given by:
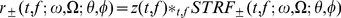
(1)where 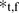 denotes convolution in time and frequency and 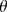 and 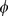 are the characteristic phases of the STRF's which determine the degree of asymmetry in the time and frequency axes respectively. The model filters 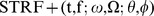 filters can be decomposed in each quadrant (upward + or downward −) into 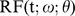 into 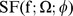 corresponding to rate and scale filters respectively. Details of the design of the filter functions 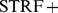 can be found in [58]. The present study uses 11 spectral filters with characteristic scales [0.25, 0.35, 0.50, 0.71, 1.00, 1.41, 2.00, 2.83, 4.00, 5.66, 8.00] (cycles/octave) and 11 temporal filters with characteristic rates [4.0, 5.7, 8.0, 11.3, 16.0, 22.6, 32.0, 45.3, 64.0, 90.5, 128.0] (Hz), each with upward and downward directionality. All outputs are integrated over the time duration of each note. In order to simplify the analysis, we limit our computations to the magnitude of the cortical output 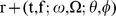 (i.e. responses corresponding to zero-phase filters).

Finally, dimensionality reduction is performed using tensor singular-value decomposition [59]. This technique unfolds the cortical tensor along each dimension (frequency, rate and scale axes) and applies singular value decomposition on the unfolded matrix. We choose 5 eigenscales, 4 eigenrates and 21 eignefrequencies resulting in 420 features with the highest eigenvalues, preserving 99.9% of the variance in the original data. The motivation for this cutoff choice is presented later.

### Cortical receptive fields

Data used here was collected in the context of a number of studies [60]–[62] and full details of the experimental paradigm are described in these publications. Briefly, extracellular recordings were performed in 15 awake non-behaving domestic ferrets (*Mustela putorius*) with surgically implanted headposts. Tungsten electrodes (3–8 MΩ) were used to record neural responses from single and multi-units at different depths. All data was processed off-line and sorted to extract single-unit activity.

Spectro-Temporal Receptive fields (STRF) were characterized using TORC (Temporally-Orthogonal Ripple Combination) stimuli [63], consisting of superimposed ripple noises with rates between 4–24 (Hz) and scales between 0 (flat) and 1.4 peaks/octave. Each stimulus was 3 sec with inter-stimulus intervals of 1–1.2 sec, and a full set of 30 TORCs was typically repeated 6–15 times. All sounds were computer-generated and delivered to the animal's ear through inserted earphones calibrated in-situ. TORC amplitude is fixed between 55–75 dB SPL.

STRFs were derived using standard reverse correlation techniques, and a signal-to-noise ratio (SNR) for each STRF was measured using a bootstrap technique (see [63] for details). Only STRFs with SNR≥2 were included in the current study, resulting in a database of 1110 STRFs (average 74 STRFs/animal). Note because of the experimental paradigm, STRFs spanned a 5-octave range with low frequencies 125, 250 or 500 Hz. In the current study, all STRFs were aligned to match the frequency range of musical note spectrograms. Since all our spectrograms start at 180 Hz and cover 5.3 octaves, we scaled and shifted the STRF's to fit this range.

The neurophysiological STRFs were employed to perform the timbre analysis by convolving each note's auditory spectrogram z(t,f) with each STRF in the database as in Equation (2).
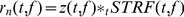
(2)The resulting firing rate vector 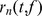 was then integrated over time yielding an average response across the tonotopic axis. The output from all STRFs were then stacked together, resulting in a 142080 (128 frequency channels ×1110 STRFs) dimensional vector. We reduced this vector using singular value decomposition and mapped it onto 420 dimensions, which preserve 99.9% of the data variance in agreement with dimensionality used for model STRFs.

### Timbre classification

In order to test the cortical representation's ability to discriminate between different musical instruments, we augmented the basic auditory model with a statistical clustering model based on support vector machines (SVM) [39]. Support vector machines are classifiers that learn a set of hyperplanes (or decision boundaries) in order to maximally separate the patterns of cortical responses caused by the different instruments.

Each cortical pattern was projected via Gaussian kernel to a new dimensional space. The use of kernels is a standard technique used with support vector machines, aiming to map the data from its original space (where data may not be linearly separable) onto a new representational space that is linearly separable. This mapping of data to a new (more linear space) through a the use of a kernel or transform is commonly referred to as the “kernel trick” [39]. In essence, kernel functions aim to determine the relative position or similarity between pairs of points in the data. Because the data may lie in a space that is not linearly separable (not possible to use simple lines or planes to separate the different classes), it is desirable to map the data points onto a different space where this linear separability is possible. However, instead of simply projecting the data points themselves onto a high-dimensional feature space which would increase complexity as a function of dimensionality, the “kernel trick” avoids this direct mapping. Instead, it provides a method for mapping the data into an inner product space without explicitly computing the mapping of the observations directly. In other words, it computes the inner product between the data points in the new space without computing the mapping explicitly.

The kernel used here is given by
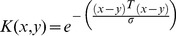
(3)where 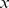 and 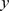 are the feature vectors of 2 sound samples. The parameter for the Gaussian kernel and the cost parameter for the SVM algorithm were optimized on a subset of the training data.

A classifier 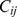 is trained for every pair of classes 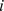 and 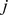. Each of these classifiers then gives a label 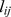 for a test sample. Note that 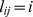 or 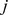. We count the number of labels 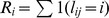. The test sample is then assigned to the class with maximum count given by 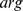 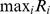. The parameter 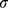 in Equation (3) was chosen by doing a grid search over a large parameter span in order to optimize the classifier performance in correctly distinguishing different instruments. This tuning was done by training and testing on a subset of the training data. For model testing, we performed a standard k-fold cross validation procedure with k = 10 (90% training, 10% testing). The dataset was divided into 10 parts. We then left out one part at a time and trained on the remaining 9 parts. The results reported are the average performance over all 10 iterations. A single Gaussian parameter was optimized for all the pair-wise classifiers across all the 10-fold cross validation experiments.

### Analysis of support vector distribution

In order to better understand the mapping of the different notes in the high-dimensional space used to classify them, we performed a closer analysis of the support vectors for each instrument pair *i* and *j*. Support vectors are the samples from each class that fall exactly on the margin between class *i* and class *j*, and therefore are likely to be more confusable between the classes. Since we are operating in the ‘classifier space’, each of the support vectors is defined in a reduced dimensional hyperspace consisting of 5 eigen-scales, 4 eigen-rates, and 21 eigen-frequencies as explained above (a total of 420 dimensions). The collection of all support vectors for each class *i* can be pulled together to estimate a high-dimensional probability density function. The density function estimate was derived using a histogram method by partitioning the sample space along each dimension into 100 bins, counting how many samples fall into each bin and dividing the counts by the total number of samples. We label the probability distribution for the *d*-th dimension (d = 1,..,420) 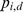. We then computed the symmetric KL divergence, 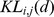 [43], between the support vectors for classes 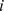 and 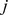 from the classifier 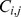 as shown in Equation (4). The KL divergence is simply a measure of difference between pairs of probability distributions, is defined is next:
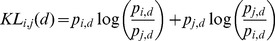
(4)The bins with zero probability were disregarded from the computation of the KL divergence. An alternative method that smoothed the probability distribution over the zero bins was also tested and yielded virtually comparable results. Overall, this analysis is meant to inform about the layout of the timbre decision space. We analyzed the significance of the results between the broad timbre classes (winds, percussions and strings) by pooling individual comparisons between instruments within each group (See Figure 5).

#### Dataset

We used the RWC music database [57] for testing the model. 11 instruments were used for this task, which included string (violin, piano, cello), percussion (vibraphone, marimba) and wind instruments (saxophone, trumpet, trombone, clarinet, oboe, and bassoon). We extracted an average of 1980 notes per instrument ranging over different makes of the instruments, as well as a wide range of pitches and styles of playing (staccato, vibrato, etc.). The notes were on average 2.7 sec in duration but varied between 0.1–18 sec. The sampling frequency of the wave files in the database was 44.1 kHz. We performed preprocessing on the sound files by first down sampling to 16 kHz then filtering using a pre-emphasis filter (FIR filter with coefficients 1 and −0.97).

### Human vs. model correlation

We tested the auditory model's ability to predict human listeners' judgment of musical timbre distances. Just like the timbre classification task, we used the cortical model augmented with Gaussian Kernels. In order to optimize the model to the test data, we employed a variation of the Gaussian kernel that performs an optimized feature embedding on every data dimension. The kernel is defined as follows:
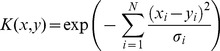
(5)where N is the number of dimensions of the features x and y. 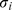
's are parameters for the kernel that need to be optimized. We define an objective function that optimizes the correlation between the human perceptual distances and the distances in the embedded space.
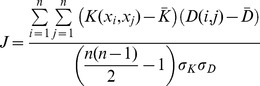
(6)where 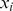 is the average profile for the i^th^ instrument over all notes; D(i,j) is the average perceived distance between the i^th^ and j^th^ instrument based on psychoacoustic results 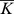 and 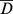 are the average distances from the kernel and the psychoacoustic experiment respectively. 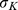 represents the variance of the kernel distances over all samples (all instrument pairs). Similarly 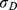 is the variance of the human perceived distances. We used a gradient ascent algorithm to learn 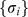 which optimize the objective function.

The correlation analysis employed the same dataset used for the human psychophysical experiment described above. Each note was 0.25 s in duration with sampling rate 44.1 kHz and underwent the same preprocessing as mentioned earlier. The absolute value of the model output was derived for each note and averaged over duration following a similar procedure as the timbre classification described above. The cortical features obtained for the three notes (A3, D4, G#4) were averaged for each instrument i to obtain 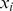. Similarly the perceived human distances between instrument i and j were obtained by averaging the (i,j)^th^ and (j,i)^th^ entry in the human distance matrix over all the 3 notes to obtain D(i,j).

Finally, the human and model similarity matrices were compared using the Pearson's correlation metric. In order to avoid overestimating the correlation between the two matrices (the two symmetric values appearing twice in the correlation), we correlated only the upper triangle of each matrix.

#### Dimensionality reduction of cortical features

As is the case with any classification problem in high-dimensional spaces, all analyses above had to be performed on a reduced number of features which we obtained using tensor singular value decomposition (TSVD), as described earlier. This step is necessary in order to avoid the curse of dimensionality which reduces the predictive power of the classifier as the dimensionality increases [64]. In order to determine the ‘optimal’ size of the reduced features, we ran a series of investigations with a range of TSVD thresholds. The analysis comparing the correlation between the cortical model and human judgments of timbre similarity is shown in Figure 8. The analysis led to the choice of 420 dimensions as near optimal. It is important to note that our tests were not fine-grained enough in order to determine the exact point of optimality. Moreover, this choice is only valid with regards to the data at hand and classifier used in this study, namely a support vector machine. If one were to choose a different classifier, the optimal reduced dimensionality may be different. It is merely a number that reflects the tradeoff between keeping a rich dimensionality that captures the diversity of the data; while reducing the dimensionality in order to fit the predictive power of the classifier.

To further emphasize this point, we ran a second analysis contrasting the system performance with the full cortical model (joint spectro-temporal modulations) against a model with separable modulations; all while maintaining the dimensionality of the reduced space fixed. This experiment (Figure 8 – red curve) confirmed that the original space indeed biases the system performance, irrespective of the size of reduced data. Results from Table 2 are also overlaid in the same figure for ease of comparison.

### Control experiments

#### i) Auditory spectrum analysis

The auditory spectrum was obtained by analyzing the input waveform with the 128 cochlear filters described above, and integrating over the time dimension. The resulting feature vector was 128×1representation of the spectral profile of each signal. Unlike a simple Fourier analysis of the signal, the cochlear filtering stage operated on a logarithmic axis with highly asymmetric filters.

#### ii) Separable spectro-temporal modulation analysis

For an input spectrogram 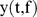, the response of each rate filter (RF) and scale Filter(SF) was obtained separately as follows:
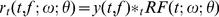
(7)

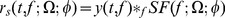
(8)where 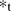 denotes convolution in time, 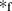 denotes convolution in frequency and 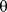 is the characteristic phase of the RF and φ is the characteristic phase of the SF which determine the degree of asymmetry in the time and frequency axis respectively. Details of the design of the filter functions 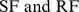 can be found in [58]. Unlike the analysis given in Equation (1), the spectral and temporal modulations were derived separately using one-dimensional complex-valued filters (either along time or along frequency axis). The resulting magnitude outputs from Equations (7–8) were then stacked together to form the feature vector with 4224 (11scales×128frequecies+22rates×128frequecies) dimensions. The dimensionality was then reduced to 420 using tensor singular value decomposition retaining 99.9% of the variance.

#### iii) Global modulation analysis

For this experiment, we used the 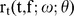 and 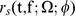 from Equations (7–8), and integrated the output over time and frequency for each note. The resulting rate and scale responses were then stacked together to form the feature vector.

#### iv) Under sampled joint modulation

In this experiment we aimed to make the dimensionality of the cortical model comparable to the separable model by under sampling the rate, scale and frequency axes. The auditory spectrogram was down sampled along the frequency axis by a factor of 2. This auditory spectrogram representation was then analyzed by 6 spectral filters with characteristic scales [0.25, 0.47, 0.87, 1.62, 3.03, 5.67] (cycles/octave) and 5 temporal filters with characteristic rates [4.0, 8.0, 16.0, 32.0, 64.0] (Hz), each with upward and downward directionality resulting in a 3840 dimensional representation. The dimensionality was then reduced to 420 using tensor singular value decomposition retaining 99.99% of the variance
