# Correction: Music in Our Ears: The Biological Bases of Musical Timbre Perception

**DOI:** 10.1371/annotation/d8b290d3-32b7-4ded-b315-d1e699bb34da

**Published:** 2013-10-08

**Authors:** Kailash Patil, Daniel Pressnitzer, Shihab Shamma, Mounya Elhilali

It has come to our attention that figures corresponding to Figure 5 and Figure 7 in the article have been switched.

References to Figure 5 in the text refer to graphics from Fig. 7 but legend from Fig. 5.

References to Figure 7 in the text refer to graphics from Fig. 5 but legend from Fig. 7.

